# Fate of the L5-S1 Disc After Thoracolumbar Fusion Ending at L5: A >5-Year Magnetic Resonance Imaging (MRI) Follow-Up Study

**DOI:** 10.7759/cureus.117582

**Published:** 2026-10-07

**Authors:** Joeffroy Otayek, Gilles Guerin, Adrien Lampilas, Pierre Emmanuel Moreau, Stéphane Wolff, Guillaume Riouallon

**Affiliations:** 1 Orthopedics and Traumatology, Hôpital Paris Saint-Joseph, Paris, FRA; 2 Orthopedics and Traumatology, Lebanese American University Medical Center, Beirut, LBN

**Keywords:** adult spinal deformity, intervertebral disc degeneration, l5-s1 disc, mri evaluation, thoracolumbar fusion

## Abstract

Purpose: This retrospective cohort study aimed to evaluate the outcomes of long thoracolumbar fusions ending at L5, focusing on revision rates and the long-term evolution of the L5-S1 disc on magnetic resonance imaging (MRI) at a minimum five-year follow-up.

Overview of literature: The decision of whether to stop a long thoracolumbar fusion at L5 or extend it to the sacrum remains highly debated due to concerns regarding adjacent segment degeneration and the subsequent need for revision surgery.

Methods: A retrospective review included 12 patients who underwent thoracolumbar fusion ending at L5 between 2007 and 2018. L5-S1 MRI Pfirrmann grades were assessed preoperatively and at the final follow-up. Oswestry Disability Index (ODI) scores, as well as pre- and postoperative radiographic spinopelvic parameters, were also recorded.

Results: Revision surgery with extension of the fusion to the sacrum was required in 41.6% of patients. Among the non-reoperated patients, 57% demonstrated progression of L5-S1 disc degeneration. The need for revision surgery was most frequently associated with preoperative sagittal imbalance.

Conclusions: Stopping a long thoracolumbar fusion at L5 may not be appropriate for patients requiring significant sagittal correction, particularly in older individuals.

## Introduction

Adult spinal deformity remains one of the most challenging conditions to manage in spine surgery. Despite advances in instrumentation and alignment strategies, determining the optimal distal fusion level continues to be debated, particularly when choosing between stopping the construct at L5 and extending it to the sacrum and pelvis [1,2]. Several studies and meta-analyses have shown that spinopelvic fixation allows for superior correction of pelvic parameters and reduces the risk of distal junctional failure [3]. However, extending the fusion to the sacrum is associated with increased rates of proximal junctional kyphosis, pseudarthrosis at L5-S1, and hardware failure [4-6]. Moreover, sacropelvic fixation may increase stiffness and reduce lumbar spine motion, potentially affecting patient function. To date, no consistent difference in long-term clinical outcomes has been demonstrated between constructs ending at L5 and those extending to the pelvis.

One theoretical concern when stopping a long thoracolumbar fusion at L5 is accelerated degeneration of the adjacent L5-S1 disc due to increased mechanical stress. Progressive disc degeneration at this level may lead to distal junctional pathology, lumbosacral pain, or radiculopathy. To our knowledge, no prior study has provided magnetic resonance imaging (MRI)-based assessment of L5-S1 disc changes with long-term follow-up.

The primary objective of this study was to characterize the long-term evolution of the preserved L5-S1 disc using MRI, at a minimum five-year follow-up, in patients who underwent thoracolumbar fusion ending at L5. A secondary objective was to descriptively evaluate the occurrence of distal construct failure requiring revision, as well as functional outcomes and postoperative spinopelvic alignment.

## Materials and methods

This is a single-center, retrospective observational study conducted in the Orthopedic Surgery Department of Hôpital Paris Saint-Joseph, Paris, France. The study was approved by Groupe Ethique Recherche Médicale (GERM) of Hôpital Paris Saint-Joseph (approval number: 00012157). All adult patients who underwent thoracolumbar fusion ending at L5 between January 2007 and December 2018 were reviewed. Patients with diverse surgical indications, including adult spinal deformity, trauma, and post-infectious instability, were included in the analysis. We grouped these varied indications together because they all share a critical biomechanical feature: the remaining mobile L5-S1 segment endures substantial stress below a long, rigid thoracolumbar construct, irrespective of the patient's initial pathology. Furthermore, aggregating these cases was necessary to assemble a meaningful cohort given the rarity of this specific surgical construct. This inclusion criteria reflects a pragmatic, real-world exploratory cohort of a relatively uncommon distal fusion level. Patients were excluded if follow-up was incomplete, specifically those without a preoperative MRI or, in the case of non-reoperated patients, those without a postoperative MRI obtained at least five years after surgery. Epidemiological and surgical variables, including patient sex, age, Lenke classification of deformity, and Cobb angle of the major curve, were collected.

The primary outcome was evaluated by the need for reintervention and the MRI evaluation of the L5-S1 disc. Any subsequent reoperation extending the construct to the sacrum, regardless of the indication, was counted as failure of the L5-S1 segment. In these cases, MRI evaluation of the disc was not feasible due to the presence of instrumentation and fusion at that level. For non-reoperated patients, a control MRI obtained at least five years postoperatively was reviewed, and follow-up duration was calculated from the date of this imaging. Because the study period spanned over a decade, MRI examinations were acquired on various 1.5-T and 3.0-T scanners according to standard institutional care protocols. While specific sequence parameters and slice thicknesses varied, disc degeneration was graded preoperatively and at the final follow-up using the five-stage Pfirrmann classification uniformly on standard sagittal T2-weighted sequences [7]. Modic endplate changes were recorded and classified as type I, II, or III [8].

Secondary outcomes included clinical and radiographic parameters. The functional outcome was assessed at the latest follow-up using the Oswestry Disability Index (ODI) [9], expressed as a percentage of disability. Radiographic evaluation was based on full-spine standing EOS images obtained preoperatively, immediately postoperatively, at the final follow-up, and, when applicable, after reoperation. On each EOS image, sagittal alignment parameters were measured, including pelvic incidence (PI), pelvic tilt (PT), sacral slope (SS), and segmental lordosis at L5-S1. The PI-lumbar lordosis (LL) mismatch was calculated and interpreted according to the Scoliosis Research Society (SRS)-Schwab classification, where PT >20° and PI-LL >10° indicate suboptimal sagittal alignment [10,11]. The occurrence of new or progressive spondylolisthesis at L5-S1 was also documented.

Owing to the small sample size, statistical comparisons were not performed; the analysis was descriptive in nature and aimed to report observed trends rather than establish statistical associations. The quantitative values are expressed as mean (minimum-maximum).

## Results

Demographics

Among the 17 initially eligible patients, four were lost to follow-up, and one was excluded because of missing preoperative MRI data, leaving 12 patients for final analysis. The cohort included two men and 10 women, with a mean age of 71 years (58-87). The mean follow-up duration was nine years (5-14). Nine patients were operated on for adult spinal deformity, two were operated on for fractures on an ankylosed spine with preservation of L5-S1 motion or multiple vertebral fractures requiring extended fixation, and one was operated on for post-infectious instability following spondylodiscitis. Among patients treated for deformity, Lenke classifications were as follows: two type IVC, six type VC, and one type VIC curves. The demographics of the patients are presented in Table 1.

**Table 1:** Demographics of the included patients MRI: magnetic resonance imaging

Patient flow	
Initially eligible patients	n=17
Lost to follow-up	n=4
Excluded (missing preoperative MRI)	n=1
Final cohort for analysis (N)	n=12
Demographics and follow-up	
Mean age, years (range)	71 (58-87)
Sex (female/male)	10/2
Mean follow-up duration, years (range)	9 (5-14)
Initial diagnosis (n)	
Adult spinal deformity	n=9
Fractures (ankylosed spine/multiple vertebral fractures)	n=2
Post-infectious instability (spondylodiscitis)	n=1
Lenke classification (deformity patients; n=9)	
Type IVC	n=2
Type VC	n=6
Type VIC	n=1

Revision surgeries

Five of the 12 patients (41.6%) underwent revision surgery with extension of fusion to the sacrum. The mean interval to reoperation was 26 months (6-44). The mean follow-up among non-reoperated patients was 120 months (60-168), exceeding the mean time to reoperation. The characteristics and causes of revision of reoperated patients are presented in Table 2.

**Table 2:** Characteristics of the reoperated patients ASD: adult spinal deformity

Case	Age at initial surgery	Sex	Initial diagnosis	Initial construct	PI (°)	LL (°)	Pfirrmann grade after initial surgery	Time to reoperation	Cause of reoperation	Reoperation construct
1	55	F	ASD; main curvature T12-L4: 47°	T6-L5	68	55	2	3 years	Pseudarthrosis at L2-L3 and L4-L5	T6-ilium
2	66	F	ASD; main curvature L2-L5: 36°	T5-L5	73	58	3	6 years	Lumbar pseudarthrosis	T5-ilium
3	64	F	ASD; main curvature T12-L5: 59°	T5-L5	71	19	3	3 years	Distal junctional failure	T2-ilium
4	69	M	Spondylodiscitis L2-L3	T11-L5	50	32	5	5 years	Distal junctional failure	T10-ilium
5	53	M	L3 fracture on Paget disease	T11-L5	75	-3	2	1 year	L3 pseudarthrosis and distal hardware failure	T11-ilium

L5-S1 disc evolution on MRI

None of the 12 patients showed preoperative Modic changes or L5-S1 spondylolisthesis. Preoperative Pfirrmann grades [7] were as follows: grade 2 in four patients, grade 3 in six, grade 4 in one, and grade 5 in one. Representative examples of L5-S1 disc degeneration across different Pfirrmann grades are presented in Figure 1.

**Figure 1:**
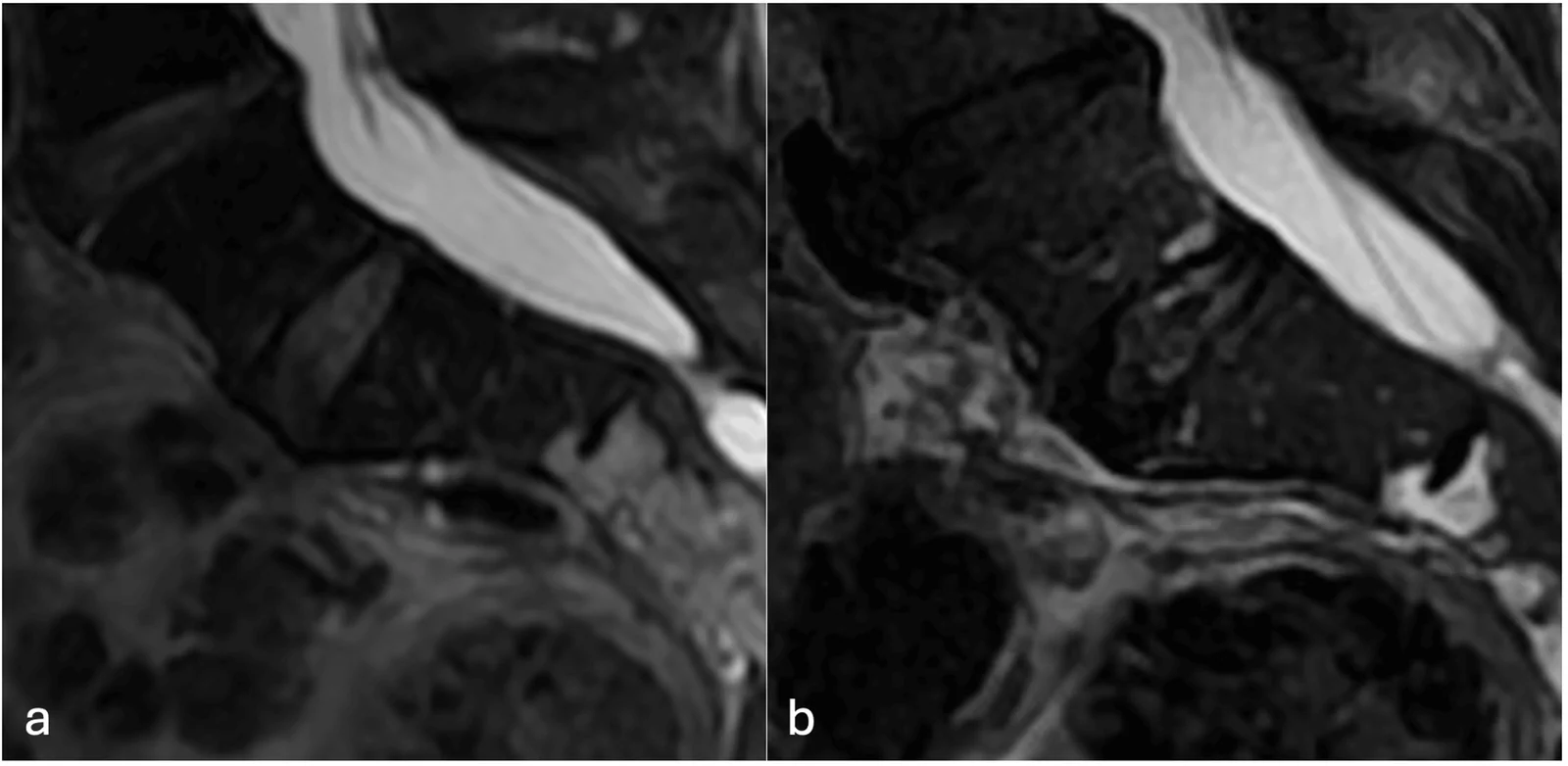
(a) Preoperative L5-S1 disc of a patient demonstrating Pfirrmann grade 2. (b) Postoperative MRI of the same patient showing progression to Pfirrmann grade 3 MRI: magnetic resonance imaging

The evolution of Pfirrmann score [7] in non-reoperated patients between the preoperative period and the last follow-up is reported in Table 3. 

**Table 3:** Evolution of Pfirrmann score in non-reoperated patients The data has been presented as n.

Preoperative Pfirrmann score	Pfirrmann score at the last follow-up					
		1	2	3	4	5
	1	-	-	-	-	-
	2	-	-	n=2	-	-
	3	-	-	n=2	n=2	-
	4	-	-	-	n=1	-
	5	-	-	-	-	-

Among the seven patients with MRI follow-up, four (57%) showed an increase of one Pfirrmann grade (e.g., grade 2→3 or 3→4), and three (43%) showed no change. No patient improved or worsened by ≥2 grades. Since postoperative MRI was not feasible in reoperated patients due to the presence of material, preoperative MRI for these patients were evaluated. Preoperative Pfirrmann scores for reoperated patients are reported in Table 4. The mean time between preoperative and postoperative MRI in reoperated patients was 10 years (5-14). No new Modic changes were observed during follow-up.

**Table 4:** Preoperative Pfirrmann score in reoperated patients The data has been presented as n.

Preoperative Pfirrmann score	Reoperated patients (n)
1	n=0
2	n=2
3	n=2
4	n=0
5	n=1

Functional outcomes

The mean ODI for the entire cohort was 31.3% (4-96). The mean ODI was 13.1% (2-40) in non-reoperated patients and 56.6% (4-96) in reoperated patients.

Radiological findings

For patients operated for deformity, the mean preoperative Cobb angle of the major curve was 45° (21-67). Preoperative, postoperative, and final follow-up values of PT, LL, and PI-LL mismatch are summarized in Tables 5-7, respectively.

**Table 5:** Pelvic tilt The data has been presented as mean (minimum-maximum).

	Preoperative, mean (min-max)	Postoperative, mean (min-max)	Pre-revision, mean (min-max)	Final follow-up, mean (min-max)
Non-reoperated patients	21 (13-33)	23 (17-37)	-	28 (20-38)
Reoperated patients	24 (14-35)	31 (21-40)	34 (27-45)	30 (19-50)
Total	22 (13-35)	26 (17-40)	-	27 (19-50)

**Table 6:** Lumbar lordosis The data has been presented as mean (minimum-maximum).

	Preoperative, mean (min-max)	Postoperative, mean (min-max)	Pre-revision, mean (min-max)	Final follow-up, mean (min-max)
Non-reoperated patients	50 (20-76)	48 (35-60)	-	43 (35-63)
Reoperated patients	36 (22-52)	36 (15-62)	32 (2-62)	32 (-3 to 58)
Total	44 (20-76)	43 (15-62)	-	38 (-3 to 63)

**Table 7:** Pelvic tilt-lumbar lordosis mismatch The data has been presented as mean (minimum-maximum).

	Preoperative, mean (min-max)	Postoperative, mean (min-max)	Pre-revision, mean (min-max)	Final follow-up, mean (min-max)
Non-reoperated patients	8 (-6 to 34)	11 (-6 to 21)	-	16 (2-30)
Reoperated patients	19 (10-52)	20 (8-49)	28 (8-60)	35 (13-78)
Total	13 (-6 to 52)	15 (-6 to 49)	-	24 (2-78)

Reoperated patients demonstrated lower LL both pre- and postoperatively compared with non-reoperated patients. All reoperated patients exhibited increased PT greater than 20° postoperatively. Preoperatively, only one non-reoperated patient had a PI-LL mismatch greater than 10°, whereas all reoperated patients had a mismatch exceeding 10° at baseline. The PI-LL mismatch remained higher postoperatively in the reoperated group. No new or progressive L5-S1 spondylolisthesis was seen on follow-up radiographs. 

## Discussion

In this series, terminating a thoracolumbar fusion at L5 resulted in a reoperation rate of 41.6% (n=5) within the first four postoperative years. Among patients who did not require reoperation, 57% (n=4) still showed progression of L5-S1 disc degeneration over a mean follow-up of 120 months (range: 60-168). Few studies have reported the results of long adult fusions to L5, and the potential for subsequent advanced L5-S1 disc degeneration is unknown [5,12]. Our findings are consistent with prior reports: Kuhns et al. observed a 35% (11/31) reoperation rate and 69% (18/26) progression of disc degeneration, often associated with sagittal imbalance [12], while Taneichi et al. reported that 50% (n=6) of patients fused to L5 required subsequent extension to the sacrum after five years, despite milder baseline deformity [2]. The characteristics and main findings of the studies in the current literature on the L5-S1 disc after long fusion to L5 are presented in Table 8.

**Table 8:** Current literature on the L5-S1 disc after long fusion to L5 MRI: magnetic resonance imaging; SAD: subsequent advanced degeneration; DJF: distal junctional failure

Study	Number of cases	Comparative study	Follow-up duration	Radiographs	MRI	Failure rate
Taneichi et al. 2020 [2]	L5: n=12; S1: n=21	Yes	Min 5y (median: 7y 2m)	Yes	No	Revision rate: L5 50% vs. S1 14%
Kuhns et al. 2007 [12]	n=31	No	Min 5y (average: 9.4y)	Yes	No	35% (11/31) reoperated, of which 23% (7/31) extension to the sacrum. 69% (18/26) SAD
Edwards et al. 2003 [13]	n=34	No	Mean 5.6y (2.1-14.3)	Yes	No	SAD 61% (19/31); revision 42% (8/19)
Edwards et al. 2004 [5]	L5: n=27; S1: n=12	Yes (matched cohort)	Average 4.8y (2-14.3)	Yes	No	SAD 67% of L5 group
Cheng et al. 2020 [14]	n=2	No	6 months	Yes	No	L5 vertebral fracture in the coronal plane
Cho et al. 2009 [15]	L5 n=24 S1 n=21	Yes	Average 3.5y (2-8)	Yes	No	SAD 58% of L5 group
Yan et al. 2018 [16]	n=67	No	Average 4.85y (2-9y)	Yes	No	L5-S1 degeneration 57.3% (36/67)
Yao et al. 2023 [1]	n=349: L4/5 n=38; S1/ilium n=311	Matching cohorts of 28	Mean 46 months	Yes	No	No significant difference in complication and revision rates
Do et al. 2024 [3]	n=76	No	Mean 3.1±1.1	Yes	No	21.1% (16/76) DJF

Our series, with a mean follow-up of nine years (5-14), presents one of the longest follow-up periods in the current literature. 

Most of the studies evaluated the L5-S1 disc based on radiographs by using Weigner grades and evaluating the subsequent advanced degeneration (SAD) [5,12,13]. To our knowledge, this is the first study to use MRI evaluation to assess the L5-S1 disc status after a fusion stopping at L5. Our study showed that preoperative Pfirrmann grade did not appear predictive of reoperation or disc degeneration progression. 

Our analysis suggests that sagittal alignment, rather than preoperative L5-S1 disc quality, is the principal factor influencing postoperative failure. Conversely, patients who underwent revision to extend fusion to the sacrum exhibited reduced lumbar lordosis both pre- and postoperatively relative to their pelvic incidence and had higher postoperative pelvic tilt. Overall, patients requiring correction of lordosis to meet SRS-Schwab criteria (postoperative PT <20° and PI-LL <10°) appear at higher risk of reoperation after a fusion ending at L5 compared with those who already satisfied these criteria preoperatively. These findings align with Do et al. [3] who identified a preoperative PI-LL mismatch greater than 40° as a strong independent risk factor for distal junctional failure in patients whose long fusion ended at L5. Their findings reinforce the concept that sagittal malalignment, rather than preoperative disc degeneration, plays a major role in postoperative failure and the need for revision. Similarly, Yan et al. reported a 57.3% rate (36/67) of subsequent L5-S1 disc degeneration after long fusion terminating at L5 in adult scoliosis [16]. They identified low pelvic incidence (<48.5°), high sagittal vertical axis (>4.4 cm), and increased sacrum-femoral distance (>5.6 cm) as independent preoperative predictors of distal disc degeneration. Edwards et al. [5] further demonstrated that sacral fixation allows better correction of sagittal balance than stopping at L5. Patients with preoperative anterior sagittal imbalance are therefore more difficult to correct when stopping at L5 and may be exposed to mechanical failure if lordosis correction is insufficient.

Interestingly, the majority of reoperations were due to mechanical factors (pseudarthrosis) rather than true adjacent segment disease at L5-S1. The 41.6% (n=5) reoperation rate for mechanical failure observed in our series is higher than that reported by Guler et al. [17] in patients with lumbopelvic fixation. This indicates that stopping at L5 does not necessarily reduce the risk of pseudarthrosis compared with sacropelvic fixation. A high rate of mechanical failure in thoracolumbar fusions ending at L5 has previously been reported, with L5 screw loosening in 16% of cases, as shown by Cho et al. [15]; in that study, mechanical failure was also associated with altered pelvic parameters. In a recent multicenter study, Yao et al. [1] compared patients undergoing fusion to L4/L5 versus those extended to S1/ilium. Although sagittal alignment restoration was initially superior in the sacropelvic group, long-term clinical outcomes, revision rates, and complication rates were comparable. These findings suggest that extending fusion to the pelvis does not necessarily guarantee improved durability or function, emphasizing the need for careful patient selection.

Although sacral fixation is a recognized risk factor for pseudarthrosis, most studies compare sacral fixation with shorter lumbar fusions and do not specifically analyze patients whose fusion stops at L5 [18-21]. Therefore, leaving one mobile segment below a long fusion may not sufficiently reduce stresses on the fused segment to prevent hardware failure. 

Functional outcomes were better in patients whose fusion stopped at L5 compared with those who underwent revision for sacropelvic fixation. However, in a comparative series of 129 patients, Martín-Buitrago et al. [22] did not find differences in quality-of-life scores between patients with and without sacropelvic fixation. It is likely that the revision surgery itself negatively affected postoperative ODI, acting as a confounding factor.

Limitations

This study has several important limitations. Primarily, the small sample size (n=12) and the single-center, retrospective design limit the generalizability of our findings. Consequently, the associations observed between altered sagittal alignment parameters and the need for revision must be interpreted as descriptive observations rather than independent predictive factors. The cohort is also characterized by heterogeneous surgical indications including adult spinal deformity, fractures, and post-infectious instability which present different biomechanical baseline risks and bone qualities. Although younger age was not an exclusion criterion, all included patients were over 55 years, limiting the applicability of our results to adolescent or young adult deformity surgery. Furthermore, larger cohorts of patients with thoracolumbar fusions ending at L5 would be useful, but such cases are uncommon; sacropelvic fixation is often preferred to reduce the risk of early adjacent segment disease.

## Conclusions

Stopping a long thoracolumbar fusion at L5 exposes patients to a high risk of mechanical failure requiring revision, with a revision rate of 41.6% (n=5) in our series. Among the non-reoperated patients, 57% (n=4) showed progression of L5-S1 disc degeneration. The preoperative quality of the L5-S1 disc, as assessed by the Pfirrmann grading system, did not appear to influence the occurrence of mechanical failure. In contrast, altered sagittal alignment parameters according to the SRS-Schwab criteria, either preoperatively or postoperatively, were more frequently associated with the need for reoperation. Leaving one mobile segment below a long thoracolumbar fusion may not sufficiently reduce mechanical stress on the construct to prevent hardware failure. Further finite element analyses comparing constructs stopping at L5 versus the sacrum would help to clarify these biomechanical differences.
